# How Orthography Modulates Morphological Priming: Subliminal Kanji Activation in Japanese

**DOI:** 10.3389/fpsyg.2016.00316

**Published:** 2016-03-30

**Authors:** Yoko Nakano, Yu Ikemoto, Gunnar Jacob, Harald Clahsen

**Affiliations:** ^1^Graduate School of Language, Communication and Culture, Kwansei Gakuin UniversityNishinomiya City, Hyogo, Japan; ^2^School of Human Welfare Studies, Kwansei Gakuin UniversityNishinomiya City, Hyogo, Japan; ^3^Potsdam Research Institute for Multilingualism, University of PotsdamPotsdam, Germany

**Keywords:** morphologically complex words, morpho-orthography, decompositon, Japanese, kanji, kana

## Abstract

The current study investigates to what extent masked morphological priming is modulated by language-particular properties, specifically by its writing system. We present results from two masked priming experiments investigating the processing of complex Japanese words written in less common (moraic) scripts. In Experiment 1, participants performed lexical decisions on target verbs; these were preceded by primes which were either (i) a past-tense form of the same verb, (ii) a stem-related form with the epenthetic vowel *-i*, (iii) a semantically-related form, and (iv) a phonologically-related form. Significant priming effects were obtained for prime types (i), (ii), and (iii), but not for (iv). This pattern of results differs from previous findings on languages with alphabetic scripts, which found reliable masked priming effects for morphologically related prime/target pairs of type (i), but not for non-affixal and semantically-related primes of types (ii), and (iii). In Experiment 2, we measured priming effects for prime/target pairs which are neither morphologically, semantically, phonologically nor - as presented in their moraic scripts—orthographically related, but which—in their commonly written form—share the same kanji, which are logograms adopted from Chinese. The results showed a significant priming effect, with faster lexical-decision times for kanji-related prime/target pairs relative to unrelated ones. We conclude that affix-stripping is insufficient to account for masked morphological priming effects across languages, but that language-particular properties (in the case of Japanese, the writing system) affect the processing of (morphologically) complex words.

## Introduction

The processing of morphologically-complex words has been subject to considerable debate in the past two decades. A core question in this area of research concerns the mechanisms the processing system employs for morphologically-complex words during word recognition. A number of studies have used the masked priming technique to examine this question. In a masked priming experiment, prime words are presented for a very short period of time only, which typically prevents the prime words from being directly recognized. Instead, masked priming is supposed to tap into subliminal processes involved in visual word recognition. A considerable number of studies have shown that native speakers can extract morphological information from inflected and derived words under masked priming conditions, by showing masked priming effects for morphologically complex word forms independently of the activation of semantic information and beyond pure orthographic priming; see Marslen-Wilson (2007). Masked morphological priming effects are supposed to be due to a morpho-orthographic segmentation mechanism that identifies the word root by stripping off affixes at an early stage of processing. Evidence for this affix-stripping mechanism comes from a series of masked priming experiments in different languages, including English (Rastle et al., 2000, 2004; Silva and Clahsen, 2008), French (Longtin and Meunier, 2005), Arabic (Boudelaa and Marslen-Wilson, 2005), Russian (Kazanina et al., 2008), German (Neubauer and Clahsen, 2009; Clahsen and Neubauer, 2010), Basque and Spanish (Duñabeitia et al., 2007). For example, Rastle et al. (2004) found that pseudo-affixed primes (e.g., *brother*), which consist of a potential but non-existent stem + affix combination (*broth-er*), prime semantically unrelated targets (e.g., *broth*) as efficiently as derivationally related prime-target pairs (e.g., *cleaner—clean*) in the masked priming paradigm. At the same time, however, primes that contain non-affixal segments (e.g., *brothel*) did not produce a corresponding (pseudo)-stem priming effect. These contrasts provide support for a decomposition mechanism by which affixes are automatically stripped off their stems, even for semantically unrelated prime-target pairs such as *brother/broth*. The presence of a pseudo-affix (e.g., -*er* in *brother*) is apparently sufficient to trigger affix stripping. Similar contrasts have also been reported for French and Russian (Longtin and Meunier, 2005; Kazanina et al., 2008).

Note, however, that all these studies come from languages with alphabetic scripts in which morphological and orthographic boundaries typically coincide. To take an example, Berg and Aronoff (submitted) demonstrate that the spelling of both inflected and derived words in English marks morphological information and that homography of suffixes and homophonous word endings tend to be avoided. For instance, while English words that end in the letters <ous> are denominal adjectives with <ous> corresponding to the suffix that derives adjectives from nouns, the same phonological sequence [əs] is spelled differently when it is not an adjective; compare, for example, [nervous]_Adjective_ with [service]_Noun_. Berg and Aronoff (submitted) show that these grapheme/morphology contingencies are not accidental, and that English spelling is not only lexically but also morphologically determined. Consequently, given the properties of this kind of writing system, affix stripping appears to be a particularly sensible strategy for word reading.

This then raises the question of whether affix stripping under masked priming conditions universally applies in morphological parsing, irrespective of a language's particular writing system. This question is still open as there are only very few studies to date that have examined masked morphological priming in languages with non-alphabetic writing systems (but see Clahsen and Ikemoto, 2012; Fiorentino et al., 2015). Against this background, the present study reports results from masked priming experiments in Japanese, a language in which morphological segmentation and orthographic boundaries sometimes fail to coincide.

## Background: some brief notes on Japanese orthography

There is a vast amount of literature on the different writing systems of Japanese, a discussion of which goes beyond the scope of the current study; see Sampson (1985, Chapter 9) for a review. Instead, this section presents a brief descriptive overview for those unfamiliar with Japanese.

Japanese has a mixed writing system consisting of *kanji*, the logographs adopted from Chinese, and *kana*, a syllable—more specifically mora-based phonographic writing system. Like the Chinese logographs, the Japanese kanji are associated with particular meanings. However, unlike the Chinese graphs (which basically have one pronunciation), kanji often allow distinct ways of pronunciation in Japanese. Kana scripts, on the other hand, are orthographic signs (“syllabograms,” Coltheart, 2014) for particular sounds, with each kana typically encoding a particular combination of a consonant and a vowel. The kana script comes in two subtypes: hiragana and katakana.

The mixed Japanese orthographical system allows users to choose whether to write a word in kanji plus kana or in kana only. Typically, words of Chinese origin and the meaningful parts of native Japanese words (i.e., the roots) are written with kanji, while grammatical morphemes (e.g., inflectional affixes) and additional elements added to the root appear in hiragana. In addition, loanwords from European languages and foreign names are entirely written with katakana. Furthermore, due to the phonographic nature of kana, it is possible (though uncommon) to write any Japanese word with kana only, even those that are normally written with the mixed script. Following Saito (1981), several experimental studies have investigated processes involved in lexical access from the different scripts; see discussion in Dehaene (2009) and Coltheart (2014). Due to its logographic nature, reading kanji engages what is labeled the “lexical” route in dual-route reading models (Coltheart et al., 2001), which provides a link from the orthographic to the semantic lexicon. By contrast, due to its phonographic nature, reading kana engages the “non-lexical” route for converting subword-level orthographic units (viz. kana) to subword-level phonological units (viz. mora). Support for this contrast comes, for example, from Chen et al.'s (2007) lexical priming experiment in which kanji targets (e.g., 展示 *tenji* “display”) were presented at short (85 ms) and long (150 ms) intervals preceded by three types of prime: (i) homophonic primes (e.g., 点字 *tenji* “braille”), (ii) semantically related primes (e.g., 陳列 *chinretu* “display”), and unrelated ones (e.g., 流浪 *ruro* “wandering”). When the prime words were presented with kanji, only the semantically related condition produced priming effects at both intervals. However, when the prime words were presented with hiragana only, the homophonic condition showed priming effects at both intervals, and the semantically related condition showed a priming effect only at the long interval. These results indicate that the lexical route is used for reading kanji and the non-lexical one for reading kana.

Regarding the spelling of morphologically complex words, it is important to note that orthographic boundaries do not always coincide with morphological segmentation in Japanese. Instead, the root-final phoneme may form a new mora with an affix or other segment, which is then spelled with a kana. Consider, for example, the consonant-final stem 眠 r /nemur/ “sleep.” When inflectional endings are added to this stem, e.g., the imperative –*e* or the past tense –*ta*, the stem-final consonant forms a mora with the suffix, which is spelled with kana. The imperative form, for example, is 眠れ “sleep!” with the kana れfor /re/. The past-tense form is 眠った “slept” with the stem-final /r/ changed to /t/ and the /tta/ segment spelled with the two kana っ and た.

## The present study

The current study examines whether morpho-orthographic decomposition (“affix-stripping”) of morphologically complex words, as reported in masked morphological priming studies of languages with alphabetic scripts, is also employed in a language with a different writing system (viz. Japanese). In previous research, priming effects under masked stimulus conditions have been reported for morphologically complex words in Japanese (Clahsen and Ikemoto, 2012; Fiorentino et al., 2015), specifically for deadjectival forms with the suffixes –*sa* and –*mi*. In both studies, significant masked priming effects were found irrespective of whether primes and targets were spelled in the common mixed script (i.e., with kanji plus kana) or in kana only. While the priming effects might indeed—as suggested by the authors of the two studies—be due to affix stripping (of -*sa* and –*mi*), parallel to the masked priming effects found for derived (and inflected) words in English and other languages with alphabetic scripts, an alternative possibility needs to be considered. This is because the critical prime-target pairs that yielded significant facilitation of target recognition times in Clahsen and Ikemoto's and in Fiorentino et al.'s studies—when written with the common mixed script—share the same kanji. Consequently it is conceivable that the reported priming effects are due to this overlap in kanji, rather than (or perhaps on top of) affix stripping. Consider, for example, the prime-target pair 楽しい - 楽しみ “delightful-delightfulness” from Fiorentino et al. (2015, Table 1) from which the orthographic overlap in terms of shared kanji is obvious. Clahsen and Ikemoto (2012) tried to reduce this kind of direct orthographic overlap between primes and targets by presenting their stimuli in the kana-only script, with the primes and the targets in hiragana. If, however, these stimuli are written in the normal (mixed) script, the prime-target pairs that yielded priming effects in their experiment share the same kanji and the ones that do not show priming have different kanji. Indeed, Clahsen and Ikemoto (2012) found the same priming patterns in a follow-up experiment in which all items were presented in the mixed script (with kanji) as in their main experiment with kana-only stimulus presentation. Given these findings, it is conceivable that activation of the mixed script including the corresponding kanji cannot be completely blocked, even when reading the prime-target pairs in the unusual kana-only script.

**Table 1:** **Mean lexical decision times (and standard deviations) by condition and prime type**.

	**/i/ form**	**-*****ta*** **form**	**Semantic overlap**	**Phonological overlap**
Identity	631 (68)		638 (76)	651 (72)
Test	646 (94)	660 (92)	700 (73)	715 (88)
Unrelated	741 (102)		731 (90)	727 (61)

To further elucidate the nature of masked priming effects of complex words in written Japanese, the present study addresses two questions. Experiment 1 asks whether decomposition of complex words under masked priming conditions is genuinely morphological (viz. “*affix* stripping”) or whether non-affixal material is also segmented from the root in Japanese. Experiment 2 asks how masked priming effects in Japanese are modulated by the particular properties of its mixed writing system, specifically how the activation of kanji affects visual word recognition.

## Experiment 1: affix stripping in Japanese masked priming

Affix stripping is a powerful morphological parsing mechanism that the masked priming technique is supposed to tap into. Stanners et al. (1979) explained morphological priming effects as follows: “…the base verb and suffix are partitioned prior to memory access and the base verb is then directly accessed” (p. 403). In other words, when a word form such as *walked* is presented as a prime, the affix –*ed* is stripped off, thereby isolating the base stem which then directly facilitates recognition of a target word such as *walk*. Crucially, non-affixal segments of morphologically unrelated words have been shown not to produce masked priming effects in English and other languages with alphabetic scripts; see Marslen-Wilson (2007). In English, for example, *darkness* primes *dark*, but *example* does not prime *exam*, reflecting the fact that (unlike <ness>) the letters <ple> do not function as an affix (Heyer and Clahsen, 2015).

In the present masked priming experiment, we examined whether this contrast also applies to Japanese. Two types of critical prime words were tested: (i) inflected –*ta* suffixed past-tense verb forms and (ii) words with the non-affixal word-final segment /i/. Both critical types of prime words have parallel surface forms with one segment added to the stem. However, while prime type (i) is a morphologically structured word form with the past-tense suffix –*ta*, prime type (ii) is the non-affixal infinitive form of consonant-final verbal stems, the most common type of verbal stem in Japanese. Crucially, the /i/ segment added to these verbal stems represents a case of phonological epenthesis (enforced by the CV phonotactics of Japanese), rather than an inflectional or derivational suffix (Kiyose, 1971; Shirota, 1998; Tagawa, 2012); see examples (1) and (2) below. The target forms used for both prime types were non-past forms of the same verbs, which consist of the same stems as the primes plus the invariant non-past affix –*u*; see example (3). With these conditions we can directly compare priming from affixed vs. non-affixal word forms on the same target words. Two additional conditions were added to assess the potential contribution of semantic and phonological relatedness. The first condition consisted of primes and targets that were semantically related. They were either synonyms (e.g., ほめる *homeru* “compliment”—タタエル *tataeru* “praise”); see examples (4) and (5) or semantic associates (e.g., あるく *aruku* “walk”—ハシル *hashiru* “run”); see examples (6) and (7). The second condition constituted primes and targets that were phonologically related, i.e., similar-sounding but otherwise unrelated prime-target pairs, e.g., たたむ *tatamu* “fold”—タタカウ *tatakau* “fight”; see examples (8) and (9). Assuming that under masked priming conditions affixed word forms are morphologically decomposed (“affix stripping”) in Japanese, we would expect to find a reliable morphological priming effect for –*ta* forms, i.e., prime type (i), but not for the non-affixal /i/ forms of prime type (ii), parallel to the masked priming results reported for English and other languages with alphabetic scripts. Furthermore, under the assumption (e.g., Rastle et al., 2000, 2004) that masked priming effects are due to morpho-orthographic segmentation (viz. affix stripping), we do not expect the semantic and phonological control conditions to yield any reliable priming effects.

(1) Prime type (i): –*ta* form, e.g., *nemutta* “slept” written with kana only and kanji plus kana

**Table:** 

	stem			suffix
International Phonetic Alphabet	ne	mɯ^ᵝ^	t	ta
Hiragana	ね	む	っ	た
Kanji (root) + Kana	眠		っ	た

(2) Prime type (ii): /i/ verb form, e.g., *nemuri* “sleep” written with kana only and kanji plus kana

**Table:** 

	stem				epenthesis
International Phonetic Alphabet	ne	mɯ^ᵝ^	ɾ^J^		i
Hiragana	ね	む		り	
Kanji (root) + Kana	眠			り	

(3) Target form: non-past verb form, e.g., *nemuru* “sleep” written with kana only and kanji plus kana

**Table:** 

	stem				suffix
International Phonetic Alphabet	ne	mɯᵝ	ɾ		ɯ^ᵝ^
Katakana	ネ	ム		ル	
Kanji (root) + Kana	眠			る	

(4) Prime type: non-past verb form, e.g., *homeru* “compliment” written with kana only and kanji plus kana

**Table:** 

	stem				suffix
International Phonetic Alphabet	ho	me	ɾ		ɯ^ᵝ^
Hiragana	ほ	め		る	
Kanji (root) + Kana	誉	め		る	

(5) Target form: non-past verb form, a synonym, e.g., *tataeru* “praise” written with kana only and kanji plus kana

**Table:** 

	stem			suffix
International Phonetic Alphabet	ta	ta	e	rɯ^ᵝ^
Katakana	タ	タ	エ	ル
Kanji (root) + Kana	讃		え	る

(6) Prime type: non-past verb form, e.g., *aruku* “walk” written with kana only and kanji plus kana

**Table:** 

	stem		suffix
International Phonetic Alphabet	a	ɾɯ^ᵝ^	kɯ^ᵝ^
Hiragana	あ	る	く
Kanji (root) + Kana	歩		く

(7) Target form: non-past verb form, a semantic associate, e.g., *hashiru* “run” written with kana only and kanji plus kana

**Table:** 

	stem				suffix
International Phonetic Alphabet	ha	ʃi	ɾ		ɯ^ᵝ^
Katakana	ハ	シ		ル	
Kanji (root) + Kana	走			る	

(8) Prime type: non-past verb form, e.g., *tatamu* “fold” written with kana only and kanji plus kana

**Table:** 

	stem				suffix
International Phonetic Alphabet	ta	ta	m		ɯ^ᵝ^
Hiragana	た	た		む	
Kanji (root) + Kana	畳			む	

(9) Target form: phonologically-related non-past verb form, e.g., *hashiru* “run” written with kana only and kanji plus kana

**Table:** 

	stem			suffix
International Phonetic Alphabet	ha	ta	ka	ɯ^ᵝ^
Katakana	タ	タ	カ	ウ
Kanji (root) + Kana	戦			う

### Participants

Twenty-eight Japanese speakers [mean age: 22 (*SD*: 6.73), age range: 18–45, 16 females and 12 males] were recruited from the undergraduate and graduate communities at Kwansei Gakuin University in Japan. All participants had normal or corrected-to-normal vision. This study was carried out in accordance with the recommendations of the Grant-in-Aids for Scientific Research of the Japan Society for the Promotion of Science, with written informed consent from all subjects. All subjects gave written informed consent in accordance with the Declaration of Helsinki.

### Materials

We constructed 24 experimental item sets, with each set consisting of four prime-target pairs. Each set was based on one of 24 Japanese verbs with consonant-final stems, with the non-past form of the respective verb (e.g., *nemuru*) serving as the target word in all four prime-target pairs of the respective item set; see Appendix A in Supplementary Material for the complete set of items. The target was preceded by one of four different primes: (i) the non-affixal /i/ form of the given verb stem, (ii) the corresponding past-tense form with the inflectional suffix –*ta*, (iii) a matched unrelated control prime, or (iv) an identity control prime in which the target word occurred as both prime and target. Priming effects were determined by comparing the mean RTs for the target words following /i/ and –*ta* primes to those following unrelated control primes. The identity control condition was added as a manipulation check of whether participants were sensitive to any properties of the prime words at all under masked presentation conditions. If this is the case, we would expect to find a repetition priming effect for Identity primes relative to Unrelated primes, i.e., reduced target RTs for the former relative to the latter.

Word-form frequencies of the items for the two prime types (i) and (ii) were matched as closely as possible using Amano and Kondo's (2003) frequency dictionary, which contains more than 340,000 words collected from a Japanese newspaper between 1985 and 1998. The mean word-form frequencies (per million) were 13.6 for the –*ta* and 19.8 for the /i/ forms, a non-significant difference [*t*_(23)_ = 1.624, *p* = 0.118] (–*ta* vs. /i/). Unrelated primes were also matched in length (mora) and with respect to word-form frequency to the targets [length: *t*(23) = 1.282, *p* = 0.213, frequency: *t*(23) < 1]. As prime types (i) and (ii) are semantically, orthographically and phonologically related to their target forms [see (1) to (3) above], the potential contributions of these properties need to be considered. To reduce potential effects of orthographic relatedness between primes and targets, we used the two distinct moraic scripts of Japanese, hiragana, and katakana. All prime words were presented in hiragana whereas all targets were presented in katakana; this is illustrated in (1) to (3) for the different prime and target forms of the verbal stem *nemur*-. Two additional conditions were added to assess the potential contribution of semantic and phonological relatedness. In the semantic control condition, there were 24 semantically-related prime-target pairs, 12 consisting of synonyms (e.g., ほめる *homeru* “compliment”-タタエル *tataeru* “praise”) and 12 semantic associates (e.g., あるく *aruku* “walk”-ハシル *hashiru* “run”). These item pairs were selected from an offline rating task in which an additional group of 22 native Japanese speakers (none of whom participated in the main experiment) rated the semantic relatedness of 96 word pairs on a 7-point scale. The 24 pairs selected for the main experiment received significantly higher semantic relatedness ratings than the unrelated control pairs [means: 5.1 (*SD*: 0.71) vs. 1.8 (*SD*: 0.7), *t*_1_(21) = 14.665, *p* < 0.001; *t*_2_(46) = 15.568, *p* < 0.001], which confirmed the semantic relatedness of the test items. The second control condition consisted of phonologically related, i.e., similar-sounding but otherwise unrelated prime-target pairs, e.g., たたむ *tatamu* “fold”-タタカウ *tatakau* “fight.” There were again 24 item sets in this condition. For the semantic and the phonological control conditions, related and unrelated primes were matched for length (in terms of mora) and for word-form frequency (all *t*s < 1).

The experimental items from the 72 item sets were distributed across four different presentation lists according to a Latin-square design, with each presentation list containing exactly one prime/target pair from each item set. As a result, each participant saw each target word only once, ensuring that no participant made repeated lexical decisions on the same target word. We added 328 filler items, resulting in a total of 400 trials per presentation list. In order to make the lexical-decision task meaningful, the target words in 200 of the 328 fillers were non-words. Thus, within each presentation list, 200 targets words were existing words, and the other 200 were non-words. Non-words were created by changing one or two mora of an existing word. The order of items was pseudo-randomized, ensuring that experimental items did not appear adjacent to each other.

### Procedure, data scoring, and analysis

Each trial started with a fixation point appearing for 500 ms in the middle of the screen, followed by a 500 ms blank screen, after which a forward mask was presented for 500 ms. Prime words were presented immediately after the mask, and remained on screen for 50 ms. At the offset of the prime, the corresponding target word was presented for 1000 ms. The next trial started 500 ms after the response or timeout. Participants were instructed to make a lexical decision to the target word by pressing one of two buttons as quickly and accurately as possible. The experiment started with a practice session with 10 items. During the experiment, three breaks were provided after every 100 trials. The presentation of the stimuli and the measurement of the reaction times were controlled by the DMDX software package (Forster and Forster, 2003). The whole experiment lasted ~20–30 min.

Timeouts (response times above 2500 ms) and trials with incorrect lexical decisions were excluded from further analyses of the reaction time data. These criteria led to the removal of 13.1% of the trials from the */i/* and 9.9% from the −*ta* condition (the numerical difference in exclusion rates between the /i/ and –*ta* conditions was non-significant, *t*_*1/2*_ < 1), with 16.1% from the semantic overlap and 22.1% from the phonological overlap condition. These exclusion rates are higher than usual for lexical-decision tasks with native speakers, but note that the participants performed lexical decisions on target words written in an unusual script.

In addition, lexical decision times which were more than 2.5 *SD*s above or below the overall participant mean were considered outliers and therefore also removed; this affected 3.4 % of all –*ta* and /i/ trials, 2.1% of all “semantic” overlap trials and 3.0 % of all “phonological” trials.

### Results

Mean lexical decision times and standard deviations by prime type and condition are shown in Table 1. Note that because /i/ and –*ta* primes were tested on the same targets, Unrelated and Identity primes had the same RTs in these two conditions.

As a manipulation check of whether participants were able to retrieve any information from the masked hiragana-spelled primes at all, we first tested for repetition-priming effects by comparing target RTs for Identity vs. Unrelated primes. Paired-samples *t*-tests revealed significant repetition priming effects for all three conditions [/i/–*ta* condition: *t*_1_(27) = 5.29, *p* < 0.001; *t*_2_(23) = 5.57, *p* < 0.001; semantic overlap: *t*_1_(27) = 4.93, *p* < 0.001; *t*_2_(23) = 5.75, *p* < 0.001; phonological overlap: *t*_1_(27) = 5.40, *p* < 0.001; *t*_2_(23) = 4.43, *p* < 0.001] suggesting that participants were sensitive to properties of the hiragana-spelled prime words despite the fact that they were written in an unusual script and were masked with only a 50 ms presentation time.

The critical test of our hypotheses requires comparisons between the Test and Unrelated conditions. One-way ANOVAs comparing RTs for targets following /i/, –*ta*, and Unrelated primes revealed a significant main effect of Prime Type [*F*_1_(2, 54) = 18.37, *p* < 0.001; *F*_2_(2, 46) = 11.34, *p* < 0.001]. To explore the source of this effect, we determined priming effects separately for /i/ and –*ta* primes. Paired-samples *t*-tests revealed a difference between the Test and Unrelated conditions for both the -*ta* prime [*t*_1_(27) = 4.51, *p* < 0.001; *t*_2_(23) = 3.57, *p* = 0.002] and the /i/ prime conditions [*t*_1_(27) = 5.376, *p* < 0.001; *t*_2_(23) = 4.352, *p* < 0.001]. With regard to the semantic and phonological control conditions, paired samples *t*-tests revealed a significant difference between the Test and Unrelated prime types for the semantic overlap condition [*t*_1_(27) = 2.49, *p* < 0.05; *t*_2_(23) = 2.17, *p* < 0.05], but not for the phonological overlap condition [*t*_1_(27) = 1.00, *p* = 0.326; *t*_2_(23) = 0.90, *p* = 0.379]^1^.

In sum, Experiment 1 revealed significant priming effects for –*ta* forms, /i/ forms, and for the semantic overlap condition, but no priming effect for the phonological overlap condition. In comparison with previous masked priming studies of languages written in alphabetic scripts, this data pattern is unusual in two ways. First, our results show a significant priming effect for semantically related prime-target pairs, suggesting that in Japanese the processor was able to access semantic information from the primes under masked priming conditions, unlike what has been reported in most previous studies on masked morphological priming which have not found any reliable semantic priming effects and have claimed that the particular early stage of processing tapped by masked priming is semantically blind; see Marslen-Wilson (2007) and Davis and Rastle (2010) for a discussion. Second, inflectionally related prime words with the past-tense suffix -*ta* were found to reliably facilitate target recognition, as was found in languages with alphabetic scripts, so were word forms with the non-affixal segment /i/, with a similar magnitude as the –*ta* forms, indicating that morphological decomposition (viz. “affix stripping”) appears to be insufficient to explain the observed priming effects.

Consider a number of alternative possibilities to explain the data pattern obtained in Experiment 1. First, as we found reliable priming in the semantic control condition, it is conceivable that priming in the critical –*ta* and /i/ conditions might also be semantic in nature, since primes and targets are semantically related in these conditions. This would be in line with claims made by Feldman and collaborators who argued that semantic information from complex words can be accessed under masked priming conditions (e.g., Feldman et al., 2009, 2015). Note, however, that the magnitudes of priming for the two critical conditions were considerably larger than for the semantic control condition (95 and 81 ms for the former vs. 31 ms for the latter; see Table 1) indicating that semantic relatedness cannot fully explain the facilitation effect obtained from –*ta* and /i/ forms. Instead, each prime-target pair in the two critical conditions contains two word forms of the same lemma. Hence, lexical identity (after morphological analysis) seems to be the crucial source of the critical priming effects.

A second possibility might be that languages differ in how complex words are segmented. While affix stripping appears to be a powerful mechanism for visual word recognition of complex words in English, for example, processing complex words in Japanese might not rely on affix stripping. Instead, the system may directly search for possible roots when reading complex words in Japanese (“root spotting”). Given this mechanism, the type of segment that a potential root is combined with—whether or not it is an affix—is irrelevant. Since roots are shared between primes and targets in both the –*ta* and the /i/ conditions, root spotting may explain our finding that the –*ta* and the /i/ conditions produced priming effects of a similar magnitude. Root spotting may also account for the priming effect we found for semantically related prime-target pairs, which was, however, reduced relative to the magnitude of the priming effect obtained for the two critical conditions. This contrast could be due to the fact that in the –*ta* and the /i/ conditions, the prime and the target words share the same root, whereas in the semantic control condition the prime and the target words had different but semantically related roots. Note, however, that while a root-spotting mechanism might be operative in reading complex words in Japanese, the masked morphological priming results for English, French, and other languages cannot be explained in these terms. Recall that in English, for example, pseudo-morphologically related prime-target pairs such as *brother-broth* yielded significant masked priming effects, while prime/target pairs such as *brothel-broth* did not, although root spotting would have predicted the root *broth* to be easily identifiable from both primes, *brother* and *brothel*, which should have yielded parallel facilitation effects. This was not the case, however. It is thus possible that root-based decomposition is a language-specific property of Japanese.

A third possibility is based on the particular properties of the Japanese writing system. Recall that primes and targets in Experiment 1 were presented in different kana scripts (primes in hiragana, and targets in katakana), and were thus not directly related, neither visually nor orthographically. If the words had been presented in the mixed script with kanji, however, the corresponding kanji versions of the primes and targets in both the –*ta* and /i/ conditions would have shared the same kanji. For illustration, consider the two primes and the target in (1) to (3) above. When written in the mixed script, both primes (the –*ta* and the /i/ forms) share the same initial Kanji [viz. 眠 in (1) to (3)] with their target word forms. Hence, if Japanese readers do not completely block the (more common) mixed script, even when reading words with kana only, they will activate the shared kanji in both the primes and the target words. This may then cause the observed facilitation effect for prime words with –*ta* and /i/. In this way, (indirect) kanji activation might be a third possible source for the observed priming effects. Experiment 2 was designed to further elucidate the sources of masked priming in Japanese.

## Experiment 2: accessing kanji in reading moraic script

Experiment 2 was designed to distinguish between the different accounts described above. Specifically, we measured priming effects for prime/target pairs that were presented- as in Experiment 1- with kana only. However, even though the prime/target pairs for Experiment 2 were unrelated in every single aspect they shared kanji if spelled with the mixed script. Consider for illustration the prime-target pair in (4), with (4a) showing both prime and target in kana only (as they appeared on screen in Experiment 2) and (4b) for the corresponding mixed script version:

(4)   a. とおり - ツウ toori ‘street’ - tsuu ‘expert’
      b. 通り - 通

Given that the critical prime-target pairs in Experiment 2 are unrelated, except for the shared kanji (e.g., 通 in 4b), the results from this experiment should allow us to decide whether or not kanji are activated while reading Japanese words with moraic scripts. If this is the case, we would expect to find facilitated target recognition for prime-target pairs such as (4a), due to the (indirectly activated) shared kanji, relative to unrelated items in which prime and target words do not have a kanji in common. Alternative sources of priming, on the other hand, should not play a role for the prime-target pairs tested in Experiment 2, as these primes and targets are neither morphologically, semantically, orthographically nor phonologically related.

### Participants

A new group of 23 native speakers of Japanese was recruited, none of whom had participated in Experiment 1 [mean age: 31.5 (*SD*: 12.34), age range: 18–57, 15 females and 8 males]. All participants had normal or corrected-to-normal vision.

### Materials

We constructed a total of 16 experimental item sets, with each item set consisting of a Test prime-target pair and an Unrelated control pair. Within each item set, the Test pair consisted of the /i/ form of a consonant-final verbal stem (e.g., *hakobi* “process”) as prime and a monomorphemic word (e.g., *un* “luck”) as target; see Appendix B in Supplementary Material for the complete item set. Primes and targets were morphologically, semantically, and phonologically unrelated, but, when written with the mixed script, share a kanji, e.g., 運in *hakobi*-*un* (運び - 運). However, as in Experiment 1, stimulus presentation was in kana only, with the primes being presented with hiragana and the targets with katakana (e.g., はこび—ウン *hakobi -un*). The Unrelated control pair consisted of the same target word as the Test pair preceded by a matched unrelated control prime (e.g., さわぎ *sawagi* “disturbance”). Unlike the Test condition, Unrelated prime words (if spelled with the mixed script) did not share any kanji with their respective targets, e.g., *sawagi—un* (騒ぎ - 運). To determine kanji-mediated priming effects, we compared RTs to the target words following the Test primes to those following unrelated primes. Word-form frequencies of related and unrelated primes were matched as closely as possible using the jpTenTen corpus [LUW, sample] (long-unit words) in Sketch Engine (https://www.sketchengine.co.uk/), which consists of about 163 million words. The mean word-form frequencies (per million) were 11.64 for the Test prime words and 13.84 for the Unrelated prime words; the difference between the two was non-significant [*t*(15) = 0.09, *p* = 0.93]. Test primes and Unrelated primes were also matched for word-form frequency with the target words. The mean word-form frequency for the targets was 27.52, which was not significantly different from either Test primes or Unrelated primes [Test vs. Target: *t*(15) = 1.295, *p* = 0.215; Unrelated vs. Target: *t*(15) = 1.494, *p* = 0.156]. The mean length (in mora) was also matched between Test and Unrelated primes [3.19 vs. 3.31, *t*(15) = 0.565, *p* = 0.58]. We also calculated the number of shared phonemes between prime and target in the related and the unrelated conditions. The mean phonological overlap was low (0.9 for Test and 1.3 for Unrelated) and not reliably different between conditions [*t*(15) = 2.1, *p* = 0.11].

The items from the 16 item sets were distributed across two presentation lists according to a Latin-square design, with each list containing exactly one of the two prime-target pairs from each set. We also added a total of 40 filler items, 12 word-word pairs, and 28 word-nonword pairs. The word-word fillers consisted of word forms with the epenthetic word-final segment /i/ (e.g., すくい *sukui*, “saving”) and other monomorphemic nouns (e.g., ハハ *haha*, “mother”). These prime-target pairs were phonologically, morphologically, and semantically unrelated, and had different kanji when spelled in mixed script writing. The non-word filler targets also had /i/ as the final segment, but did not constitute existing words in Japanese.

### Procedure

The experimental procedure was parallel to Experiment 1. Prime words were presented for 50 ms. Incorrect responses and timeouts (2500 ms) were excluded from further analyses (6.5% of all experimental trials), with similar proportions of excluded data for the Test (6.4%) and the Unrelated conditions (6.4 vs. 6.6%, *t*_*1/2*_ < 1). Lexical decision times which were more than 2.5 *SD*s above or below the overall participant mean were considered outliers and were removed (2.8% of all test trials). In addition, the results from one participant had to be excluded from any further analysis because of a high overall error rate (50% of all critical trials). We also excluded two items because of high error rates across participants (34.8 and 39.1%).

### Results

Mean lexical decision times were 693 ms (*SD*: 76) for the Test condition and 727 ms (*SD*: 113) for the Unrelated condition, a significant difference for both participants and items [*t*_1_(21) = 2.406, *p* = 0.025; *t*_2_(13) = 2.591, *p* = 0.022].

This finding from Experiment 2 demonstrates a kanji-mediated priming effect for stimuli that were entirely written in kana only, and were otherwise unrelated. We conclude from this finding that Japanese readers activate kanji while reading words written in moraic scripts. In the following, we discuss the implications of this finding for the results of Experiment 1 and with respect to masked priming effects for complex words in Japanese more generally.

## General discussion

The current study investigated how the processing of complex words in Japanese is modulated by properties of its writing system. Experiment 1 showed significant priming effects of a similar magnitude on the same target words from both past-tense forms with the inflectionally suffix –*ta* and morphologically simplex word forms with a non-affixal (epenthetic) segment (/i/). In Experiment 2, we found a significant priming effect for prime-target pairs which were entirely unrelated except for the fact that prime and target share a kanji, if they had been spelled with the common mixed script. Recall, however, that in order to avoid any direct visual after-image and/or orthographic overlap between prime and target, the stimuli in both experiments were actually not presented with the mixed script, but were entirely written with the moraic scripts (primes in hiragana, and targets in katakana). Nevertheless, the results of Experiment 2 indicate that the processor activates the corresponding mixed-script versions of the stimuli even when processing words entirely presented with kana. Also note that indirect kanji activation is subliminal and automatic, as the primes were presented under masked priming conditions, which prevented participants from consciously recognizing the prime words. A related effect has been obtained by Thierry and Wu (2007) who observed unconscious translation effects in Chinese/English bilinguals' reading of English words.

The mechanism of “affix stripping” which has been claimed to explain masked priming effects in English and other languages with alphabetic scripts only provides a partial account of the priming patterns for Japanese in our experiments. While the priming effect obtained for –*ta* suffixed past-tense forms in Experiment 1 is consistent with affix stripping, non-affixal /i/ forms were also found to yield a priming effect of a similar magnitude as –*ta* forms, even though /i/ is an epenthetic vowel rather than a morphological affix. We also observed a significant semantic priming effect in Experiment 1, again unexplainable in terms of affix stripping. Furthermore, Experiment 2 revealed a (kanji-mediated) priming effect for prime-target pairs which were morphologically completely unrelated, another finding that affix stripping cannot account for.

A second possibility we considered was that the decomposition mechanism that operates under masked-presentation conditions is *root-driven* in Japanese, unlike, for example, in English in which it is apparently affix-driven. That is, while English readers may try to identify potential affixes to be stripped off, Japanese readers may search for potential roots when reading complex words. However, root spotting also provides only a partial account for our findings. While the similar-size priming effects for both the –*ta* and the /i/ conditions as well as the (smaller) semantic priming effect found in Experiment 1 can be attributed to overlapping or (semantically) related roots, the priming effect obtained in Experiment 2 cannot be explained in these terms, as all primes and targets tested here had unrelated roots.

**Figure 1:**
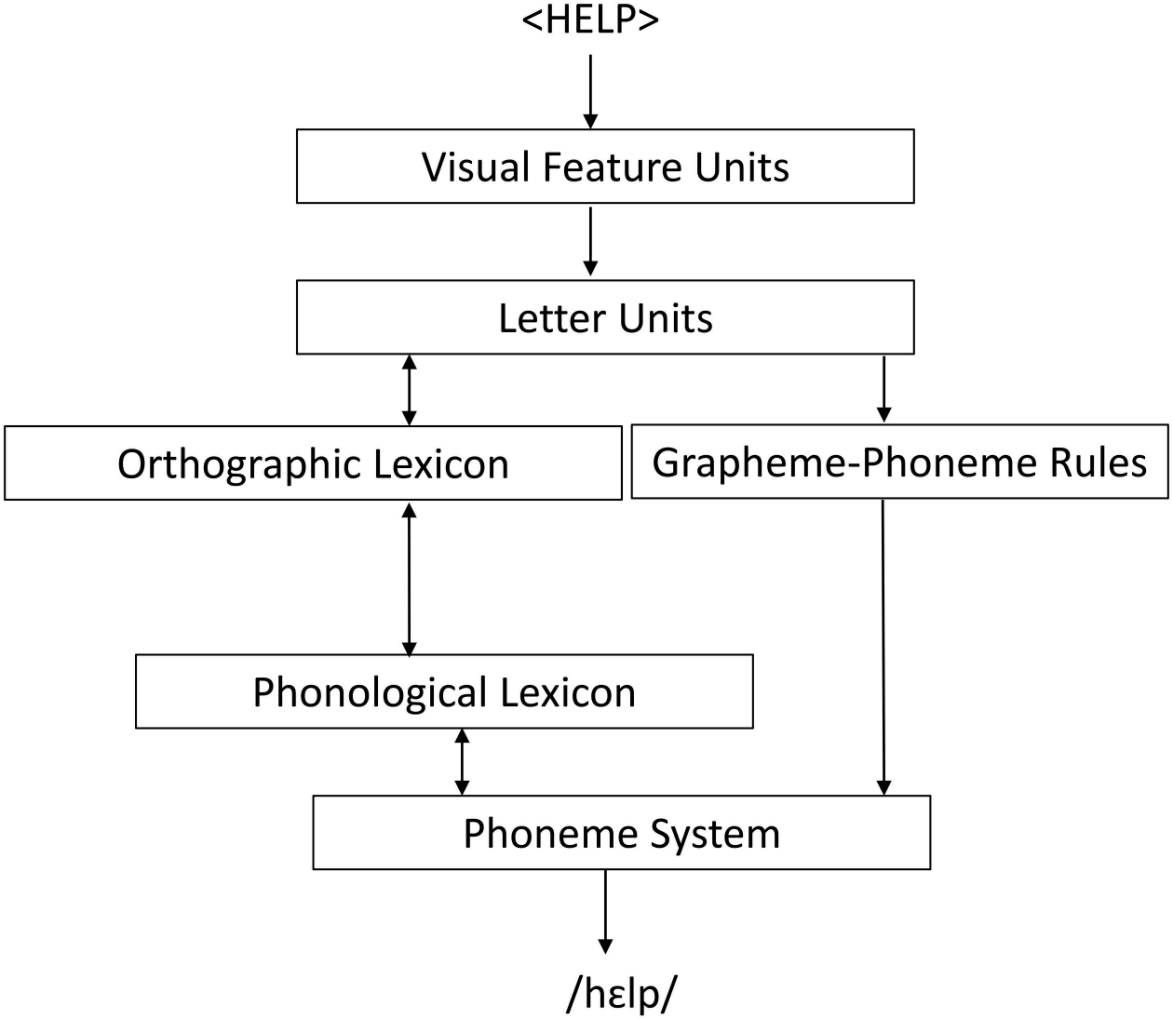
**The Dual-Route Cascaded Model (DRC); see Coltheart et al. (2001: 213)**.

This leaves us with the third possibility suggested above, that the priming effects obtained in the two experiments are orthographic in nature, due to the indirect orthographic overlap (in terms of kanji) between primes and targets. Recall, however, that all our stimuli were presented in moraic kana scripts. To see how kanji might be activated under such circumstances, consider our findings in the light of the dual-route reading model shown in Figure 1.

The DRC model assumes two alternative routes for the processing of written words, a lexical and a non-lexical one. If a stimulus is processed via the lexical route, the processor accesses the orthographic lexicon and activates a lexical entry for the word which corresponds to the orthographic properties of the particular stimulus. This entry is connected to a corresponding entry in the phonological lexicon which contains information about the phonological properties of the particular word. If a stimulus is instead processed via the non-lexical route, the processor does not access the orthographic lexicon, but instead directly activates a phonological representation of the stimulus on the basis of sign-sound correspondence rules. The DRC model has also been applied to Japanese. Coltheart (2014) proposed that in reading the common mixed script of Japanese, the lexical route is used for the logographic (kanji) components and the non-lexical route for the “syllabograms” of the two moraic scripts. A DRC account of Japanese reading and writing has also been used to explain why Japanese patients with aphasia are often selectively impaired for kana, with performance for kanji remaining intact (e.g., Sasanuma and Fujimura, 1972; Sasanuma, 1975). As can be seen from Figure 1, while the DRC account posits two distinct pathways for reading, the lexical and the non-lexical routes, these two routes are indirectly linked through the phoneme system and the phonological lexicon. Subliminal kanji activation as found in our experiments can be explained through these links. Consider one of our prime words spelled with hiragana and suppose that this is read via the non-lexical reading route. The hiragana syllabograms then activate corresponding phonemes in the “phoneme system” and these, through the links shown in Figure 1, activate entries in the phonological lexicon and subsequently the orthographic lexicon. As entries in the Japanese orthographic lexicon also contain information about how a given phonological string is spelled with kanji, the corresponding kanji are also (albeit indirectly) activated. In this way, a kanji-mediated priming effect may arise even for stimuli entirely presented in moraic scripts.

Indirect kanji activation may not only account for why affixal and non-affixal prime words produced priming effects of a similar magnitude in our Experiment 1, but may also shed new light on findings from previous masked morphological priming studies on Japanese. Both Clahsen and Ikemoto (2012) and Fiorentino et al. (2015) found significant priming effects for word forms with both the productive (–*sa*) and the unproductive (–*mi*) nominalization suffix, which they interpreted in terms of affix stripping. Note, however, that the critical prime-target pairs used in both these studies had the same kanji overlap as the –*ta* and /i/ forms tested in the current Experiment 1. Indeed, in Fiorentino et al. (2015), primes and targets were even written in the mixed script (with kanji) when presented on screen. Thus, the priming effects reported in these studies are not necessarily morphological in nature, but may also be explainable through indirect (in the case of Clahsen and Ikemoto, 2012) or direct (in the case of Fiorentino et al., 2015) kanji activation.

Finally, we also found a significant semantic priming effect in Experiment 1, an unusual finding given previous studies which have typically not obtained semantic priming effects under masked priming conditions. It is conceivable that the semantic priming effect we found for Japanese is also a reflection of its particular writing system, in the following way. Recall that the mixed script (with kanji) is the common way of reading and writing in Japanese, and that kanji are logograms which represent words or roots and engage the lexical reading route; see also Perfetti et al. (2007) for evidence suggesting that logograms activate distinct brain reading networks. Coltheart (2014) notes that kanji are ‘indivisible wholes that are not composed of subword-level orthographic elements’. Arguably, the processor can directly retrieve semantic information from such logograms. As Japanese readers are used to reading through this lexical route, semantic information might be more directly and perhaps more quickly accessible for them than readers of alphabetic scripts who are more used to reading via the non-lexical route. We acknowledge, however, that this final consideration remains speculative and that further research is needed to determine how different writing systems affect semantic effects under masked priming conditions.

In conclusion, the experimental results reported here should not be taken to mean that the Japanese language comprehension system does without morphological decomposition or without affix stripping. Instead, our results on Japanese, in comparison to those on English and languages with other alphabetical scripts, suggest that language-particular properties, in the present case differences between their writing systems, modulate the way morphologically complex words are processed during reading.

## Author contributions

YN/YI: Substantial contributions to the acquisition, analysis, or interpretation of data for the work; Drafting the work or revising it critically for important intellectual content; Final approval of the version to be published; Agreement to be accountable for all aspects of the work in ensuring that questions related to the accuracy or integrity of any part of the work are appropriately investigated and resolved. GJ/HC: Substantial contributions to the conception or design of the work; Drafting the work or revising it critically for important intellectual content; Final approval of the version to be published; Agreement to be accountable for all aspects of the work in ensuring that questions related to the accuracy or integrity of any part of the work are appropriately investigated and resolved.

## Funding

Alexander-von-Humboldt Professorship (HC), Scientific Grant-in-Aids (C) (No. 24520484, Yoko Nakano) and Kwansei Gakuin University Research Grant (A) (YN). We also acknowledge the support of the Deutsche Forschungsgemeinschaft and Open Access Publishing Fund of University of Potsdam for covering Frontiers' publishing charges.

### Conflict of interest statement

The authors declare that the research was conducted in the absence of any commercial or financial relationships that could be construed as a potential conflict of interest.

## Footnotes

^1^As regards corrections for multiple comparisons, note that although the design included three prime types, the Identity primes only served as a manipulation check to see whether the experimental technique (viz. masked priming) works with our Japanese stimuli and was not critical to test any of our hypotheses. Consequently, the phonological and semantic control conditions only consist of two critical prime types and therefore do not require any multiple comparison correction. On the other hand, the analyses for the morphological item set involve comparisons between three conditions (/*i*/, –*ta*, and unrelated). However, the differences between these conditions were robust enough even after Bonferroni-correction.
