# Supplementary material for: How Orthography Modulates Morphological Priming: Subliminal Kanji Activation in Japanese
Source: Front Psychol. 2016 Mar 30;7:316. doi: 10.3389/fpsyg.2016.00316 (PMC4811944; doi:10.3389/fpsyg.2016.00316)
Supplement: Supplementary file 1 [file DataSheet1.docx]

**Appendix A: Experiment 1**

For each item set, the appendix shows primes and targets as they appeared on screen (i.e. primes in hiragana, targets in katakana) along with their corresponding Roman–alphabet versions (in parentheses), and their corresponding mixed-script versions. The meanings of unrelated primes and targets are in single-quotation marks.

| /i/ prime | -*ta* prime | Unrelated prime | | Target |  |
| --- | --- | --- | --- | --- | --- |
| さそい | さそった | しぶい | ‘astringent’ | サソウ | ‘invite’ |
| (sasoi) | (sasotta) | (shibui) |  | (sasou) |  |
| 誘い | 誘った | 渋い |  | 誘う |  |
|  |  |  |  |  |  |
| かたむき | かたむいた | にがい | ‘bitter’ | カタムク | ‘slant’ |
| (katamuki) | (katamuita) | (nigai) |  | (katamuku) |  |
| 傾き | 傾いた | 苦い |  | 傾く |  |
|  |  |  |  |  |  |
| ねむり | ねむった | ほそい | ‘slim’ | ネムル | ‘sleep’ |
| (nemuri) | (nemutta) | (hosoi) |  | (nemuru) |  |
| 眠り | 眠った | 細い |  | 眠る |  |
|  |  |  |  |  |  |
| おどろき | おどろいた | せまい | ‘narrow’ | オドロク | ‘be surprised’ |
| (odoroki) | (odoroita) | (semai) |  | (odoroku) |  |
| 驚き | 驚いた | 狭い |  | 驚く |  |
|  |  |  |  |  |  |
| あつまり | あつまった | うすい | ‘thin’ | アツマル | ‘gather’ |
| (atsumari) | (atsumatta) | (usui) |  | (atsumaru) |  |
| 集まり | 集まった | 薄い |  | 集まる |  |
|  |  |  |  |  |  |
| まとまり | まとまった | とうとい | ‘precious’ | マトマル | ‘cohere’ |
| (matomari) | (matomatta) | (tootoi) |  | (matomaru) |  |
| 纏まり | 纏まった | 尊い |  | 纏まる |  |
|  |  |  |  |  |  |
| にごり | にごった | すずしい | ‘cool’ | ニゴル | ‘get muddy’ |
| (nigori) | (nigotta) | (suzushii) |  | (nigoru) |  |
| 濁り | 濁った | 涼しい |  | 濁る |  |
|  |  |  |  |  |  |
| うるおい | うるおった | まぶしい | ‘bright’ | ウルオウ | ‘moisten’ |
| (uruoi) | (uruotta) | (mabushii) |  | (uruou) |  |
| 潤い | 潤った | 眩しい |  | 潤う |  |
|  |  |  |  |  |  |
| さぐり | さぐった | あまい | ‘sweet’ | サグル | ‘search’ |
| (saguri) | (sagutta) | (amai) |  | (saguru) |  |
| 探り | 探った | 甘い |  | 探る |  |
|  |  |  |  |  |  |
| つまずき | つまずいた | みじかい | ‘short’ | ツマズク | ‘stumble’ |
| (tsumazuki) | (tsumazuita) | (mijikai) |  | (tsumazuku) |  |
| 躓き | 躓いた | 短い |  | 躓く |  |
|  |  |  |  |  |  |
| まよい | まよった | さむい | ‘cold’ | マヨウ | ‘hesitate’ |
| (mayoi) | (mayotta) | (samui) |  | (mayou) |  |
| 迷い | 迷った | 寒い |  | 迷う |  |
|  |  |  |  |  |  |
| あじわい | あじわった | おそい | ‘late’ | アジワウ | ‘taste’ |
| (ajiwai) | (ajiwatta) | (osoi) |  | (ajiwau) |  |
| 味わい | 味わった | 遅い |  | 味わう |  |
|  |  |  |  |  |  |
| うたがい | うたがった | みずうみ | ‘lake’ | ウタガウ | ‘doubt’ |
| (utagai) | (utagatta) | (mizuumi) |  | (utagau) |  |
| 疑い | 疑った | 湖 |  | 疑う |  |
|  |  |  |  |  |  |
| かざり | かざった | たらい | ‘basin’ | カザル | ‘decorate’ |
| (kazari) | (kazatta) | (tarai) |  | (kazaru) |  |
| 飾り | 飾った | 盥 |  | 飾る |  |
|  |  |  |  |  |  |
| たのみ | たのんだ | かすみ | ‘mist’ | タノム | ‘ask’ |
| (tanomi) | (tanonda) | (kasumi) |  | (tanomu) |  |
| 頼み | 頼んだ | 霞 |  | 頼む |  |
|  |  |  |  |  |  |
| まなび | まなんだ | ひつじ | ‘sheep’ | マナブ | ‘learn’ |
| (manabi) | (mananda) | (hitsuji) |  | (manabu) |  |
| 学び | 学んだ | 羊 |  | 学ぶ |  |
|  |  |  |  |  |  |
| まもり | まもった | ころも | ‘clothing’ | マモル | ‘protect’ |
| (mamori) | (mamotta) | (koromo) |  | (mamoru) |  |
| 守り | 守った | 衣 |  | 守る |  |
|  |  |  |  |  |  |
| おこない | おこなった | たから | ‘treasure’ | オコナウ | ‘do’ |
| (okonai) | (okonatta) | (takara) |  | (okonau) |  |
| 行い | 行った | 宝 |  | 行う |  |
|  |  |  |  |  |  |
| すわり | すわった | まくら | ‘pillow’ | スワル | ‘sit’ |
| (suwari) | (suwatta) | (makura) |  | (suwaru) |  |
| 座り | 座った | 枕 |  | 座る |  |
|  |  |  |  |  |  |
| はじまり | はじまった | さむらい | ‘samurai’ | ハジマル | ‘begin’ |
| (hajimari) | (hajimatta) | (samurai) |  | (hajimaru) |  |
| 始まり | 始まった | 侍 |  | 始まる |  |
|  |  |  |  |  |  |
| おわり | おわった | ほたる | ‘firefly’ | オワル | ‘end’ |
| (owari) | (owatta) | (hotaru) |  | (owaru) |  |
| 終わり | 終わった | 蛍 |  | オワル |  |
|  |  |  |  |  |  |
| きまり | きまった | かばん | ‘bag’ | キマル | ‘decide’ |
| (kimari) | (kimatta) | (kaban) |  | (kimaru) |  |
| 決まり | 決まった | 鞄 |  | 決まる |  |
|  |  |  |  |  |  |
| ひろがり | ひろがった | まぼろし | ‘phantom’ | ヒロガル | ‘expand’ |
| (hirogari) | (hirogatta) | (maboroshi) |  | (hirogaru) |  |
| 広がり | 広がった | 幻 |  | 広がる |  |
|  |  |  |  |  |  |
| ねらい | ねらった | きずな | ‘bond’ | ネラウ | ‘aim’ |
| (nerai) | (neratta) | (kizuna) |  | (nerau) |  |
| 狙い | 狙った | 絆 |  | 狙う |  |

**Appendix B: Experiment 2**

For each item set, the appendix shows primes and targets as they appeared on screen (i.e. primes in hiragana, targets in katakana) along with their corresponding Roman-alphabet versions (in parentheses), their corresponding mixed-script versions, and their meanings (shown in single-quotation marks).

| Test prime |  | Unrelated prime | | Target |  |
| --- | --- | --- | --- | --- | --- |
| たより | ‘letter’ | あつまり | ‘gathering’ | ベン | ‘convenience’ |
| (tayori) |  | (atsumari) |  | (ben) |  |
| 便り |  | 集まり |  | 便 |  |
|  |  |  |  |  |  |
| あき | ‘vacancy’ | いつわり | ‘lie’ | ソラ | ‘sky’ |
| (aki) |  | (itsuwari) |  | (sora) |  |
| 空き |  | 偽り |  | 空 |  |
|  |  |  |  |  |  |
| はこび | ‘process’ | さわぎ | ‘noise’ | ウン | ‘luck’ |
| (hakobi) |  | (sawagi) |  | (un) |  |
| 運び |  | 騒ぎ |  | 運 |  |
|  |  |  |  |  |  |
| とおり | ‘street’ | あそび | ‘play’ | ツウ | ‘expert’ |
| (toori) |  | (asobi) |  | (tsuu) |  |
| 通り |  | 遊び |  | 通 |  |
|  |  |  |  |  |  |
| そむき | ‘rebellion’ | うつろい | ‘change’ | セ | ‘back’ |
| (somuki) |  | (utsuroi) |  | (se) |  |
| 背き |  | 移ろい |  | 背 |  |
|  |  |  |  |  |  |
| つがい | ‘pair’ | きそい | ‘competition’ | バン | ‘guard’ |
| (tsugai) |  | (kisoi) |  | (ban) |  |
| 番い |  | 競い |  | 番 |  |
|  |  |  |  |  |  |
| あきない | ‘business’ | いこい | ‘relaxation’ | ショウ | ‘quotient’ |
| (akinai) |  | (ikoi) |  | (shoo) |  |
| 商い |  | 憩い |  | 商 |  |
|  |  |  |  |  |  |
| なつき | ‘fondness’ | かかわり | ‘relation’ | フトコロ | ‘inside-pocket’ |
| (natsuki) |  | (kakawari) |  | (futokoro) |  |
| 懐き |  | 関わり |  | 懐 |  |
|  |  |  |  |  |  |
| そそぎ | ‘pouring’ | みのり | ‘crop’ | チュウ | ‘note’ |
| (sosogi) |  | (minori) |  | (chuu) |  |
| 注ぎ |  | 実り |  | 注 |  |
|  |  |  |  |  |  |
| かわり | ‘substitute’ | わらい | ‘laugh’ | ダイ | ‘generation’ |
| (kawari) |  | (warai) |  | (dai) |  |
| 代わり |  | 笑い |  | 代 |  |
| ‘substitute’ |  | ‘laugh’ |  | ‘generation’ |  |
|  |  |  |  |  |  |
| わかり | ‘comprehension’ | まなび | ‘learning’ | フン | ‘minute’ |
| (wakari) |  | (manabi) |  | (fun) |  |
| 分かり |  | 学び |  | 分 |  |
|  |  |  |  |  |  |
| いたわり | ‘appreciation’ | しぼり | ‘iris’ | ロウ | ‘labor’ |
| (itawari) |  | (shibori) |  | (roo) |  |
| 労り |  | 絞り |  | 労 |  |
|  |  |  |  |  |  |
| さからい | ‘disobedience’ | むすび | ‘knot’ | ギャク | ‘opposite’ |
| (sakarai) |  | (musubi) |  | (gyaku) |  |
| 逆らい |  | 結び |  | 逆 |  |
|  |  |  |  |  |  |
| まがり | ‘curve’ | あつかい | ‘treatment’ | キョク | ‘music’ |
| (magari) |  | (atsukai) |  | (kyoku) |  |
| 曲がり |  | 扱い |  | 曲 |  |
|  |  |  |  |  |  |
| あやつり | ‘manipulation’ | さとり | ‘enlightenment’ | ミサオ | ‘chastity’ |
| (ayatsuri) |  | (satori) |  | (misao) |  |
| 操り |  | 悟り |  | 操 |  |
|  |  |  |  |  |  |
| ながし | ‘sink’ | よろこび | ‘joy’ | リュウ | ‘style’ |
| (nagashi) |  | (yorokobi) |  | (ryu) |  |
| 流し |  | 喜び |  | 流 |  |
